# The validity and reliability of the Turkish version of the quality of recovery-15 (QoR-15) questionnaire

**DOI:** 10.1097/MD.0000000000037867

**Published:** 2024-04-19

**Authors:** Emine Aslanlar, Durmuş Ali Aslanlar, Cennet Doğanay, Özkan Önal, Mehmet Sargin, Faruk Çiçekci, Fatih Kara, İnci Kara

**Affiliations:** a Selcuk University Faculty of Medicine, Department of Anesthesiology and Reanimation, Konya, Turkey; b Necmettin Erbakan University Faculty of Medicine, Department of Medical Pharmacology, Konya, Turkey; c Antalya City Hospital, Department of Anesthesiology and Reanimation, Antalya, Turkey; d Selcuk University Faculty of Medicine, Department of Public Health, Konya, Turkey.

**Keywords:** anesthesia recovery period, patient reported outcome measures, postoperative period, surveys and questionnaires

## Abstract

Quality of recovery (QoR) is a significant component of peri-operative health status and is influenced by patients’ characteristics and surgical and anesthetic methods. The QoR-15 scale is a patient-reported outcome questionnaire that measures postoperative QoR. The validity of the QoR-15 scale has been proven in many languages. In this study, we aimed to translate the QoR-15 questionnaire into Turkish and evaluate its validity in the Turkish population. After being translated into Turkish, the questionnaire was administered to 190 patients who underwent obstetric, gynecological, orthopedic, or thoracic surgery under general or regional anesthesia. The Turkish version of QoR-15 (QoR-15T) was administered 2 times: before surgery and 24 hour after surgery. The feasibility, reliability, validity and responsiveness of the QoR-15T were evaluated. Because 13 patients were discharged within 24 hour postoperatively, the study was completed with 177 patients. The recruitment and completion rates of questionnaire were 95% and 93.1% respectively. The completing time of the questionnaire was 2.5 minutes preoperatively and 3.5 minutes postoperatively. The scale yielded a Cronbach α value of 0.75, a Cohen effect size of 1.42, and a standardized response mean of 1.39. There was a significant positive correlation (95% confidence interval; *R* = 0.68, *P* < .001) between QoR-15T and visual analog scale postoperatively. The correlation of the items with the total QoR-15T score ranged from 0.19 to 0.60. The total scores of preoperative and postoperative QoR-15T were mean: 130.67, standard deviation: 15.78 and mean: 108.23, standard deviation: 13.06, respectively, with a significant difference between them (*P* < .01). The QoR-15T is feasible, reliable, valid, and responsive among patients undergoing surgery under general and regional anesthesia.

## 1. Introduction

Postoperative recovery can be defined as patients’ regaining of preoperative functional abilities and improvement of adverse symptoms.^[1]^ The postoperative recovery process is affected by many variables, especially biological, physiological and psychological variables.^[2]^ In evaluating the postoperative recovery process, special attention is paid to patient-reported outcomes (PROs) alongside traditional clinical outcomes such as postoperative organ dysfunction, morbidity or surgical complications. PROs are measurements taken directly from the patient without any comment on the patient’s response by a clinician. These measurements draw clinicians’ attention to outcomes that matter to patients.^[3]^ There are numerous measurement tools based on PROs in the literature, such as Short-form 36, EuroQol 5-dimension questionnaire, and quality of recovery score (QoR)-40. The QoR-15 is one of these measurement tools, and the American Society for Enhanced Recovery recommends that it be administered to patients during the peri-operative period to improve the quality of health services.^[4]^

The QoR-15 was developed in 2013 as a revised and brief form of the QoR-40 to assess the QoR after surgery and anesthesia. This unidimensional scale aims to evaluate patients’ quality of recovery and well-being in the early postoperative period.^[5–7]^ It is a questionnaire consisting of 15 questions evaluating the patients physical comfort, physical independence, emotional state, psychological support, and pain status. For each question in the QoR-15, the patient is asked to rate the degree of improvement on a scale of 0 to 10. A total score between 0 to 150 is obtained after all responses have been summed. A score of 150 represents an excellent health status.^[5]^ If the total score is between 136 to 150; perfect; between 122 to 135; good; if it is between 90 to 121; medium; if it is between 0 to 89, it indicates poor recovery.^[7]^

This one-page questionnaire has been translated into different languages, such as French, German, Persian, Italian, and Korean.^[8–12]^ It has been reported in systematic reviews that different language versions of the QoR-15 have adequate validity and reliability in assessing the postoperative QoR.^[13,14]^ However, there was no Turkish version of the QoR-15 in the literature when we began the study. Therefore, we aimed to develop the Turkish version of the QoR-15 (QoR-15T) and to evaluate its psychometric properties in the Turkish population.

## 2. Methods

This prospective observational study was approved by the Local Ethics Committee of Selçuk University Medicine Faculty (11.08.2021, No: 2021/407) and it was registered at www.clinicaltrials.gov (NCT05124054). An informed written consent form was obtained from all participants. Permission was obtained from Paul Myles who developed the QoR-15 scale to translate the scale into Turkish via e-mail. According to studies on validity and reliability in the literature, the sample size should be 10 to 20 times the number of items on a scale.^[15]^ Therefore, given that the QoR-15 consisted of 15 items, we multiplied it by ten, and calculated the sample size to be at least 150.

The study’s sample group consisted of patients who underwent surgery between December 2021 and March 2022. Patients aged 18 to 65 whose native Turkish speakers, with American Society of Anesthesiologists (ASA) physical condition class I-III and planned for elective surgery were included in the study. Patients with cognitive dysfunction, psychiatric disease, alcohol addiction, or substance abuse, who were expected to need postoperative respiratory support, unable to respond to the questionnaire due to a changed mental state during the postoperative evaluation, or who developed significant postoperative complications were excluded from the study.

### 2.1. Translation and cultural adaptation of the questionnaire

**Figure 1.**
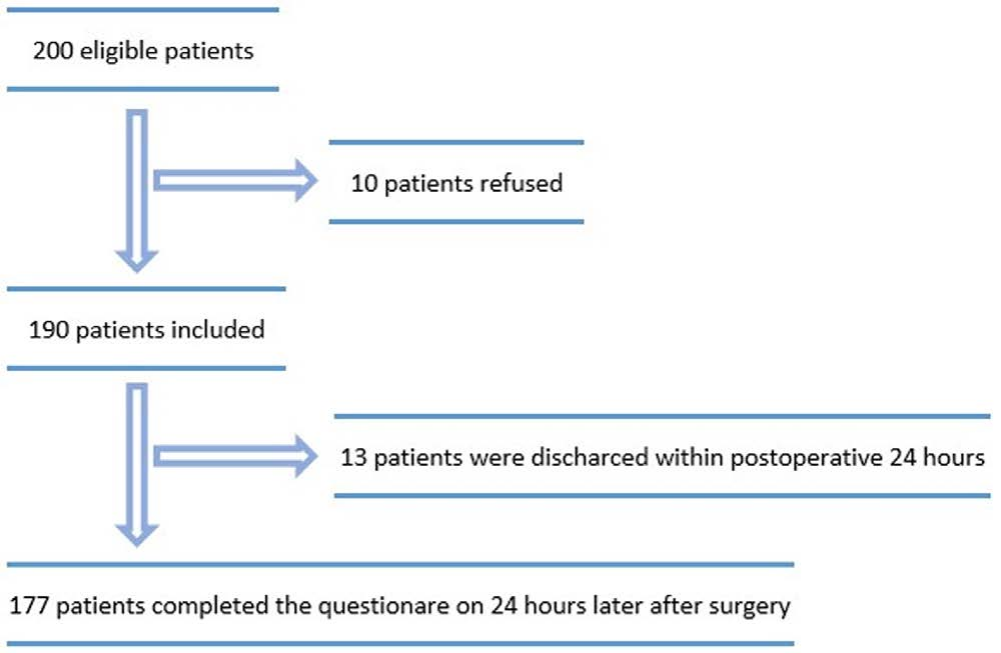
Turkish version patients flowchart.

QoR-15 was first translated into Turkish by 3 specialist physicians. After reaching a consensus on the consistency and adequacy of the meaning of the 3 different translations, the Turkish version was translated back into English by 2 linguists, one of whom is a native English speaker. Then, an expert group consisting of 3 anesthesiologists and 2 translators reached a consensus on the final version of the Turkish version (QoR-15T) (Fig. 1). It was applied to 10 pilot patients to test whether all the items in the QoR-15T were sufficiently understandable and quickly answered. After it was seen that all ten pilot patients answered each question without difficulty, the QoR-15T was finalized and applied to the study patients. No cultural adjustments were required.

### 2.2. Protocol

Age, gender, weight, height, education status, comorbidity, ASA physical status score, anesthesia and surgery type and duration, length of stay in the postanesthesia care unit, and hospital stay of the patients were recorded.

The QoR-15T scale was evaluated through rater-based interviews: The questions were read aloud to the patients by the practitioner, and the patients’ numerical responses were recorded. The questionnaire was administered to the patients 2 times, 1 day before and the first day after the surgery. As there is no gold standard scale for postoperative recovery, a 100-mm visual analog scale (VAS) was used to measure the convergent validity of the QoR-15T.^[5,8]^ On postoperative day 1, the practitioner asked patients to rate their overall recovery from 0 to 100 (0, “worst possible recovery” and 100, “best possible recovery”).

### 2.3. Psychometric validation

The primary aim was to evaluate the feasibility, reliability, validity, and responsiveness of QoR-15T 24 hours postoperatively. Acceptability and feasibility were evaluated with the recruitment rate, completion rate, and time taken to complete the questionnaire.

Reliability was assessed with internal consistency. Internal consistency was calculated as the averaged correlation between each of the questions.

Validity was assessed by comparing QoR-15T and VAS scores. Also, inter-item correlations were calculated.

Responsiveness was measured with standardized response mean (SRM) and Cohen effect size. The SRM shows how the mean of the QoR-15T scores changed in terms of the QoR-15T scores’ standard deviation (SD). The Cohen effect size is calculated by dividing the mean score difference between the preoperative and postoperative periods by the baseline SD.

### 2.4. Statistical analysis

Data were reported as mean (SD), median (inter-quartile range), or number (percentage) as appropriate.

Categorical data were presented as frequency and percentage. Differences in distribution were analyzed with the Kruskal–Wallis test. Mann–Whitney *U* test was used to compare QoR-15T scores. Correlations were calculated with Spearman correlation coefficient.

All analyses were performed using SPSS Statistics for Windows, version 21.0 (SPSS; IBM Corp, Armonk, NY). The statistical significance for all analyses was set at *P* < .05.

## 3. Results

**Figure 2.**
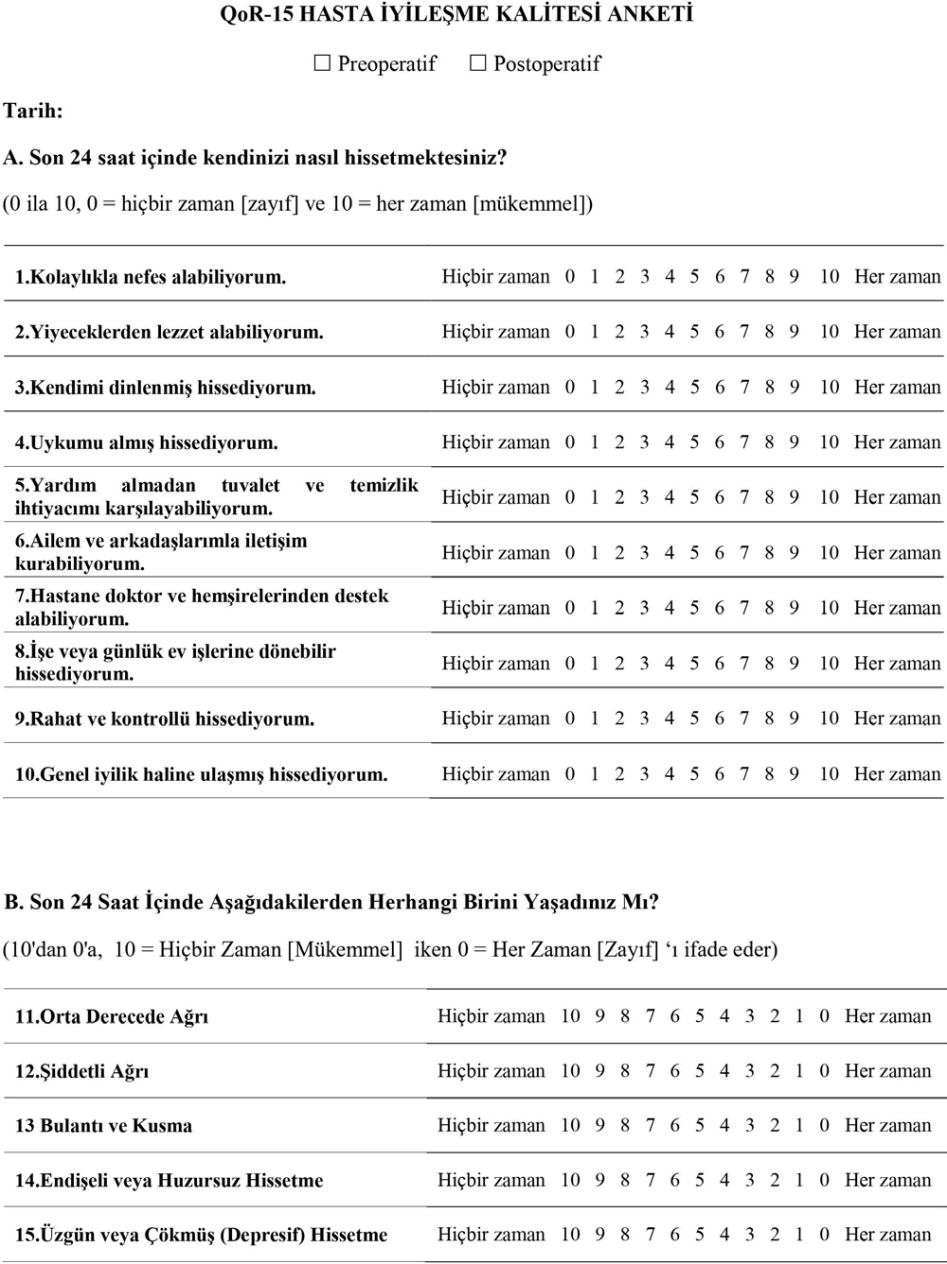
Turkish version of quality of recovery-15 questionnaire (QoR-15T).

Of the 200 patients who were planned to participate in the study, ten did not want to participate, so the recruitment rate was 95%. Because 13 patients were discharged within 24 hours postoperatively, the study was completed with 177 patients (Fig. 2). The questionnaire completion rate was 93.1% (177/190).

The mean age of the patients was 42.4 years. Of the patients, 79.7% (141) were female. The mean duration of the surgery was 92.1 minutes. Of the patients, 50.8% (90) had received regional anesthesia, and 49.2% (87) had received general anesthesia. Demographic data of the patients are presented in Table 1. All psychometric tests assessed QoR-15T performed 24 hours after surgery.

**Table 1:** Patient demographics datas (n = 177).

Age (years)	42.4 ± 15.3
Gender (male/female)	36 (20.3)/141 (79.7)
Body mass index (kg m^−2^)	29.7 ± 6.4
Duration of surgery (min)	92.1 ± 42.3
PACU (min)	23.8 ± 8.9
ASA	
I	32 (18.1)
II	126 (71.2)
III	19 (10.7)
Type of anesthesia	
General	87 (49.2)
Regional	90 (50.8)
Type of surgery	
Gynecological	53 (29.9)
Obstetrics	64 (36.2)
Orthopedic	34 (19.2)
Thoracic surgery	26 (14.7)

Number (%) or mean ± SD.

ASA = American Society of Anesthesiologists, PACU = postanesthesia care unit.

### 3.1. Acceptability and feasibility

The mean time for completing the questionnaire was 2.5 minutes preoperatively and 3.5 minutes postoperatively. The postoperative response time never exceeded 4 minutes.

### 3.2. Reliability

Internal consistency was measured using Cronbach alpha. The interitem Cronbach alpha value was 0.75.

### 3.3. Validity

#### 3.3.1. Convergent validity

Convergent validity was determined by examining the relationship between the postoperative QoR-15T and VAS, which revealed a significant positive correlation (95% confidence interval [CI]; *R* = 0.68, *P* < .001). The correlation of the items with the total QoR-15T score ranged from 0.19 to 0.60. The matrix of interitem correlation is presented in Table 2.

**Table 2:** Interitem correlation matrix for the QoR-15T.

Item no	Total score	1	2	3	4	5	6	7	8	9	10	11	12	13	14
**1**	**0.34**	–													
**2**	**0.54**	0.15	–												
**3**	**0.38**	0.13	**0.23**	–											
**4**	**0.41**	0.04	**0.39**	**0.28**	–										
**5**	**0.58**	0.10	**0.24**	**0.16**	0.12	–									
**6**	**0.19**	0.14	−0.13	−0.11	−0.04	0.05	–								
**7**	**0.34**	**0.21**	0.05	0.05	0.08	**0.28**	0.09	–							
**8**	**0.57**	0.09	**0.35**	**0.21**	**0.20**	**0.37**	0.01	**0.24**	–						
**9**	**0.59**	**0.18**	**0.25**	**0.19**	**0.17**	**0.35**	**0.26**	0.09	**0.38**	–					
**10**	**0.52**	0.06	**0.22**	0.05	0.08	**0.22**	**0.18**	**0.18**	**0.32**	**0.55**	–				
**11**	**0.53**	**0.21**	**0.29**	**0.24**	**0.20**	**0.21**	0.00	0.07	**0.19**	0.13	0.10	–			
**12**	**0.45**	**0.17**	**0.15**	−0.06	0.08	**0.22**	0.03	**0.17**	0.08	**0.17**	**0.17**	**0.20**	–		
**13**	**0.32**	0.11	0.10	0.12	0.08	**0.18**	−**0.15**	0.04	0.05	0.02	−0.08	**0.20**	**0.24**	–	
**14**	**0.60**	0.09	**0.29**	**0.19**	**0.17**	**0.23**	0.04	−0.01	**0.19**	0.10	**0.19**	**0.34**	**0.26**	**0.20**	–
**15**	**0.53**	0.02	0.11	0.04	0.03	**0.19**	0.07	0.14	0.07	**0.16**	**0.21**	**0.29**	**0.29**	**0.27**	**0.64**

Bold values are *P* < .05 or *P* < .01.

#### 3.3.2. Discriminant validity

Discriminant validity was determined by comparing VAS scores of ≥70 (good) or <70 (poor) with postoperative QoR-15T total scores. As expected, patients with a VAS score of <70 had significantly lower postoperative QoR-15T total scores (mean: 107.83, SD: 12.28) than those with a VAS score of ≥70 (mean: 115.5, SD: 7.11) (*P* < .01).

### 3.4. Responsiveness

The Cohen effect size was 1.42, and the standardized response mean was 1.39, indicating excellent responsiveness (Table 3). The total scores of preoperative and postoperative QoR-15T were mean: 130.67, SD: 15.78 and mean: 108.23, SD: 13.06, respectively, with a significant difference between them (*P* < .01) (Table 3). The variation of QoR-15T and total scores from the preoperative to the postoperative period is shown in Table 3.

**Table 3:** Changes in health status of patients before preoperative and postoperative period (24 hours).

QoR-15 item	Preoperative (mean ± SD)	Postoperative (mean ± SD)	Mean change (95% CI)	Change from baseline (%)	Cohen effect size	SRM
1. Able to breathe easily	9.28 ± 1.23	8.05 ± 1.37	−1.23 (−0.98; −1.47)	13	0.99	0.74
2. Been able to enjoy food	9.46 ± 1.29	7.51 ± 1.96	−1.94 (−1.64; −2.25)	21	1.50	0.94
3. Feeling rested	7.71 ± 2.52	5.68 ± 1.65	−2.02 (−1.62; −2.43)	26	0.80	0.74
4. Have had a good sleep	7.26 ± 2.75	5.14 ± 1.89	−2.11 (−1.65; −2.58)	29	0.77	0.68
5. Able to look after personal toilet and hygiene unaided	9.47 ± 1.63	6.23 ± 2.22	−3.24 (−2.89; −3.59)	34	1.98	1.38
6. Able to communicate with family or friends	9.84 ± 0.64	8.95 ± 1.57	−0.88 (−0.65; 1.12)	9	1.37	0.55
7. Getting support from hospital doctors and nurses	9.75 ± 0.97	9.27 ± 1.24	−0.48 (−0.31; 0.66)	5	0.50	0.41
8. Able to return to work or usual home activities	8.68 ± 2.09	5.68 ± 2.30	−3.00 (−2.56; −3.43)	35	1.43	1.02
9. Feeling comfortable and in control	8.75 ± 2.04	6.38 ± 1.90	−2.36 (−1.98; −2.75)	27	1.16	0.92
10. Having a feeling of general well-being	8.69 ± 2.01	6.53 ± 2.12	−2.16 (−1.77; −2.56)	25	1.08	0.81
11. Moderate pain	7.64 ± 2.83	5.80 ± 1.85	−1.84 (−1.37; −2.32)	24	0.65	0.58
12. Severe pain	9.27 ± 2.00	5.81 ± 1.86	−1.56 (−1.22; −1.90)	17	0.78	0.68
13. Nausea or vomiting	9.23 1.93	8.09 ± 1.45	−1.14 (−0.82; −1.45)	12	0.59	0.54
14. Feeling worried or anxious	7.46 ± 2.96	8.66 ± 2.30	1.19 (1.65; 0.72)	16	0,40	0.38
15. Feeling sad or depressed	8.15 ± 2.72	8.56 ± 2.06	−0.40 (0.80; 0.01)	5	0.15	0.15
Total score	130.67 ± 15.78	108.23 ± 13.06	−22.43 (−20.04; −24.82)	17	1.42	1.39

Mean ± SD.

Cohen effect size = mean change in score divided by the baseline (preoperative) SD.

Standardized response mean = mean change in score divided by its SD.

The postoperative QoR-15T score had a significant negative correlation with surgery duration (95% CI; r = −0.33, *P* < .001) and hospital stay duration (95% CI; r = −0.18, *P* < .02). The QoR-15T score did not correlate with patient age (95% CI; r = −0.14, *P* = .06).

There was no difference in postoperative QoR-15T scores between men (mean: 107.25, SD: 12.89) and women (mean: 108.48, SD: 13.214) (*P* = .6).

There was no significant relationship between patients’ education level and postoperative QoR-15T scores (*P* = .42).

There was no significant difference in postoperative QoR-15T total scores between those who had received general and those who had received regional anesthesia (*P* = .2).

## 4. Discussion

The study results revealed that the QoR-15T is a valid, reliable, and feasible scale for evaluating the quality of postoperative recovery following general and regional anesthesia in the Turkish population.

Pain, nausea-vomiting, weakness, and tiredness are among the most common problems in the postoperative period. In this period, in addition to using traditional clinical outcomes, it is essential to evaluate the postoperative recovery status using a measurement instrument that focuses on situations essential to the patients and is easy to use. In our study, although ten patients refused to answer the questionnaire, almost all of the patients (93%) could complete the QoR-15T within a maximum of 4 minutes at 24 hours postoperatively. These results show that the QoR-15T is a scale that can be easily used to measure the quality of postoperative recovery in Turkish culture. When a scale is adapted, convergent validity is determined based on correlation measurement instruments available in the literature for the same purposes.^[16]^ There is no gold standard measure in the literature for evaluating the QoR. Similarly to the original study, this study used VAS as an alternative measure to evaluate recovery.^[5,12]^ We assessed the convergent validity by comparing the QoR-15T scale scores with the VAS scores and found the correlation coefficient was the same as in the original study (*R* = 0.68 in the original study; *R* = 0.68 in our study). These results were interpreted as sufficient convergent validity for the QoR-15T scale.

Regarding reliability, our study reported a Cronbach alpha of 0.75 (the acceptable value for Cronbach alpha is >0.70) indicating that the QoR-15T is a reliable instrument for assessing patient recovery. Furthermore, our results are similar to those of other studies in the literature regarding the reliability of the QoR-15T (0.76–0.90).^[8,17–19]^

The QoR-15T correlated well with the VAS value, which was also used in previous studies and to assess overall recovery after surgery.^[12,18,20]^ The inter-item correlation coefficients ranged from 0.19 to 0.60, indicating little redundancy between items and each question assessing a different aspect of postoperative recovery.^[6]^

The responsiveness of QoR-15T was assessed with Cohen effect size and standardized response mean.^[21]^ Our Cohen effect size had a significant value (1.42), similar to that of Nakatani and colleagues (1.42).^[22]^ The standardized response mean was 1.39. These values indicate that the questionnaire effectively detects clinically significant changes in postoperative recovery. This level of strength suggests that the questionnaire can be used as a patient-reported measure.^[5]^

Patients completed the QoR-15T questionnaire in 2.5 minutes in the preoperative period and 3.5 minutes in the postoperative period on average, indicating that the questionnaire is a feasible instrument of measurement.

In line with previous studies, this study found no association between the QoR-15T and demographic factors such as sex, age, education level, and ASA physical status.^[5,19,23]^ This result suggests that the QoR-15 is less susceptible to these demographic variables.

Our study differs from other studies in that previous studies only evaluated patients who underwent surgery under general anesthesia. In contrast, this study evaluated patients who received both general and regional anesthesia. Patients who received regional anesthesia were expected to have higher total scores. However, our study found no difference in total QoR-15 scores between patients who received general and those who received regional anesthesia. Future studies should evaluate the anesthesia type’s impact on the quality of postoperative recovery.

Our study has some limitations: First, it could have included patients who undergone the same surgery, but evaluated patients who underwent gynecological, obstetric, orthopedic, or thoracic surgery. Second, to allow patients to respond, the questionnaire was administered with a researcher reading aloud the items in the questionnaire. If the patients had completed the questionnaire independently, the results could have been different. However, the reverse order of the items in Parts A and B (containing positive and negative items) could have caused confusion. Because, in questions about pain severity, a low score indicates less pain on the conventional pain rating scale but severe pain on the QoR-15.^[8]^ Another limitation of our study is that test-retest measurement, which is an additional method for calculating reliability, was not performed.

In conclusion, we translated the QoR-15 into Turkish and evaluated its feasibility in patients undergoing various types of surgery under general or regional anesthesia. The QoR-15T has acceptable validity, reliability, responsiveness, and applicability and can be used to measure the quality of postoperative recovery in the Turkish population.

## Author contributions

**Conceptualization:** Durmuş Ali Aslanlar.

**Data curation:** Cennet Doğanay.

**Formal analysis:** Durmuş Ali Aslanlar.

**Investigation:** Emine Aslanlar, Mehmet Sargin, Faruk Çiçekci.

**Methodology:** Emine Aslanlar, Durmuş Ali Aslanlar.

**Writing – original draft:** Emine Aslanlar, Durmuş Ali Aslanlar.

**Writing – review & editing:** Özkan Önal, Fatih Kara, İnci Kara.
